# Evaluating dimensionality reduction for genomic prediction

**DOI:** 10.3389/fgene.2022.958780

**Published:** 2022-10-14

**Authors:** Vamsi Manthena, Diego Jarquín, Rajeev K. Varshney, Manish Roorkiwal, Girish Prasad Dixit, Chellapilla Bharadwaj, Reka Howard

**Affiliations:** ^1^ Department of Statistics, University of Nebraska-Lincoln, Lincoln, NE, United States; ^2^ Agronomy Department, University of Florida, Gainesville, FL, United States; ^3^ State Agricultural Biotechnology Centre, Centre for Crop and Food Innovation, Murdoch University, Murdoch, WA, Australia; ^4^ Center of Excellence in Genomics & Systems Biology, International Crops Research Institute for the Semi-Arid Tropics (ICRISAT), Hyderabad, India; ^5^ Khalifa Center for Genetic Engineering and Biotechnology, United Arab Emirates University, Al-Ain, United Arab Emirates; ^6^ ICAR- All India Coordinated Research Project (AICRP)- Chickpea, ICAR-Indian Institute of Pulses Research (IIPR), Kanpur, Uttar Pradesh, India; ^7^ ICAR-Indian Agricultural Research Institute (IARI), New Delhi, India

**Keywords:** dimensionality reduction, chickpea, genomic selection, randomized algorithms, genomic prediction

## Abstract

The development of genomic selection (GS) methods has allowed plant breeding programs to select favorable lines using genomic data before performing field trials. Improvements in genotyping technology have yielded high-dimensional genomic marker data which can be difficult to incorporate into statistical models. In this paper, we investigated the utility of applying dimensionality reduction (DR) methods as a pre-processing step for GS methods. We compared five DR methods and studied the trend in the prediction accuracies of each method as a function of the number of features retained. The effect of DR methods was studied using three models that involved the main effects of line, environment, marker, and the genotype by environment interactions. The methods were applied on a real data set containing 315 lines phenotyped in nine environments with 26,817 markers each. Regardless of the DR method and prediction model used, only a fraction of features was sufficient to achieve maximum correlation. Our results underline the usefulness of DR methods as a key pre-processing step in GS models to improve computational efficiency in the face of ever-increasing size of genomic data.

## 1 Introduction

Plant breeding techniques have led to significant gains in crop yields for many decades. Improvements to crops were made through phenotypic and pedigree data. The use of molecular markers is a relatively new technique for improving conventional breeding strategies. Most traits that have economic and agronomic importance are quantitative in nature and are controlled by multiple genes of small effect. The advent of high-throughput genotyping and the ever-reducing cost of Single Nucleotide Polymorphisms (SNP) assays brought forward the possibility of using dense SNP arrays for the prediction of phenotypic traits. Genomic selection (GS) proposed by Meuwissen et al. (2001) is a method where the entire genome is used to predict the phenotypic traits.

A major challenge of GS lies in estimating the large number of marker effects (*p*) using information from only a few number of individuals *n*. Many models and algorithms have been proposed in the literature to overcome this challenge. Some prominent shrinkage-based models include ridge-regression BLUP, BayesA and BayesB (Meuwissen et al., 2001), LASSO (Usai et al., 2009), elastic net (Zou and Hastie, 2005), Bayesian LASSO (de los Campos et al., 2009), reproducing kernel Hilbert spaces (Gianola et al., 2006; De los Campos et al., 2010), and support vector regression (Moser et al., 2009; Long et al., 2011). While these models deal with the dimensionality issue through shrinkage methods, they did not incorporate multi-environment information.

Environment and genotype by environment (G×E) interactions strongly impact the performance of lines from one environment to the next, and hence accounting for these effects could improve the performance of the prediction models (Jarquín et al., 2017; Roorkiwal et al., 2018). Burgueño et al. (2012) included the G×E interactions information through structured genetic correlations and found that using multi-environment information improved the prediction accuracy. Jarquín et al. (2014) proposed the multiplicative reaction-norm model (MRNM), an extension to the standard G-BLUP model and an alternative to the models proposed by Burgueño et al. (2012). The MRNM models allowed the environmental effect to be included along with the genomic information and the G × E interaction effect by modeling the covariance structure. They showed that introducing interactions between the environmental covariates and molecular markers can increase the proportion of variance explained by the model as well as increase the prediction accuracy. Several other models have been proposed to take the G × E interactions into account and improve the prediction accuracies (Crossa et al., 2004, 2006; Burgueño et al., 2008, 2011; Heslot et al., 2012).

Most of these methods deal with the high-dimensional aspect of genomic selection, also known as genomic prediction, through modern shrinkage procedures. Shrinkage methods perform dimensionality reduction as a part of the modeling process. This paper examines the utility of dimensionality reduction (DR) methods as a pre-processing step to genomic prediction. In “small *n* large *d*” problems such as genomic prediction, several markers (aka variables or features) may be insignificant in explaining the phenotypic response. Thus, it is essential to eliminate such insignificant features to improve the prediction process. Eliminating irrelevant features before running prediction models could also help reduce the resource requirements for the computations of the models in terms of memory and time. Furthermore, separating the DR process from the modeling step could allow for greater flexibility in choosing models used for the GS.

The primary objective of this work is to study the utility of implementing dimensionality reduction as a pre-processing step in GS. We employed five different DR methods and investigated their ability to improve GS models. Furthermore, we compared their relative reduction abilities and studied the trends in the prediction accuracy as a function of the number of markers retained from the original marker data. To answer these objectives, we created reduced data sets with an increasing number of markers using each DR method, performed genomic prediction for each size, and computed their respective prediction accuracy values. We hypothesize that the prediction accuracy values would plateau beyond a certain size. Any further increase in the number of markers in the input data set would not significantly improve and potentially even harm the prediction accuracy.

The rest of the paper is organized as follows. First, we present the different DR methods in the materials and methods section. For each method, we also describe their implementation in creating reduced marker data sets. Next, we describe the genomic prediction models and cross-validation schemes used, along with a description of the real data set. Following that, we present the results of the DR for each method, along with comparisons. Finally, we conclude with a discussion and future directions.

## 2 Materials and methods

Traditional methods and approaches to data analysis prove unsuitable in the face of massive modern data sets. The need of the hour dictates the development of new statistical algorithms that can analyze these large data sets. The objective of these new algorithms is to work within reasonable constraints on computational resources and time while providing reliable solutions to research problems. With ever-increasing access to storage resources and a reduction in the cost of collecting additional features on observational units, the dimension of data sets is constantly increasing. For instance, the advent of high-throughput phenotyping and genotyping technologies in life sciences has led to massive data sets that present unprecedented storage and computational challenges.

High-dimensional data could be classified as “large *n*, large *d*” datasets or “small *n*, large *d*” datasets. A primary assumption in the analysis of such high-dimensional data is the existence of a lower-dimensional subspace that contains all of the important information and allows for reliable inference and prediction of the response variable. Given a matrix ***A*** ∈ ***R*** ^*n*×*d*^, obtaining a ‘compressed’ matrix that captures the most important and relevant information present in **A** has significant practical importance. The process of obtaining this compressed matrix is referred to as dimensionality reduction. Dimensionality reduction assumes greater importance in the case of computations involving high-dimensional data sets. Low-rank approximations for such matrices are commonly used in various statistical applications such as principal component analysis (PCA), k-mean clustering, data compression, solving linear equations, *etc.*

It is well known that the Singular Value Decomposition (SVD) obtains the best rank-*k* approximation of any matrix (Eckart and Young, 1936). Although SVD provides the best rank-k approximation of a matrix, it is increasingly infeasible to compute it due to the sheer size of modern data sets. Consider again the matrix ***A*** ∈ ***R*** ^*n*×*d*^ and without loss of generality, assume that *d* > *n*. The computation of the best rank-k approximation **A**_*k*_ takes *O* (*nd* ^2^) time (Golub and Van Loan, 2013), which can prove prohibitive for modern large data sets. Recent decades have witnessed substantial progress in developing several DR methods to obtain accurate low-rank representations of matrices while overcoming the computational challenges presented by SVD. These algorithms compute a low-rank approximation that can replace the original matrix in computations without loss in precision.

Dimensionality reduction methods can be categorized into three main approaches (Ghashami et al., 2015): sparsification, feature extraction, and feature selection. Sparsification refers to generating a sparse version of the matrix that can be stored efficiently and lead to faster matrix multiplication (Achlioptas and Mcsherry, 2007; Drineas and Zouzias, 2011). Linear combinations of the original features can also generate Low-rank approximations of a matrix to form new combined features. These linear combinations are determined through pre-multiplication of the original features with a coefficients matrix, and this approach is called feature extraction. Two popular algorithms for feature extraction, PCA (Pearson, 1901) and LDA (Fisher, 1936), project the data onto a lower-dimensional representation. The third approach—called feature selection—refers to a method where we find a small subset of the original features that approximate the whole set of features. Forward selection, backward selection, and best subset selection (James et al., 2013) algorithms are commonly used feature selection algorithms. Feature selection is equivalent to the column subset selection problem (CSSP) in numerical linear algebra, which has been well studied and has seen several applications in data analysis (Boutsidis et al., 2009; Deshpande et al., 2006; Drineas et al., 2012, 2006a,b, 2008, 2011; Mahoney and Drineas, 2009; Papailiopoulos et al., 2014).

The feature extraction method yields a compressed matrix formed by computing the linear combinations of the original features. While this method has been shown to provide reliable approximations to the original data matrix for further computations, there is a prominent issue in working with a combination of features. The linear combinations may not be suitable to make statistical inferences about the original data scale, and there may be no sensible interpretation of the combinations themselves in specific applications. Given this drawback of the feature extraction method, the feature selection approach to dimensionality reduction presents itself as a more suitable choice. The feature selection method involves selecting a small subset of the original features to create a compressed features matrix and avoids the issues related to inference and interpretability. For this reason, we examined the feature selection approach in greater detail by investigating four feature selection based algorithms. Each of these methods presents a fundamentally different approach to feature selection.

Dimensionality reduction methods can also be categorized as deterministic or randomized based on how the lower-dimensional representation is derived. In deterministic methods, features are selected fixedly based on some property of the data, such as the singular values, as in the case of PCA. Features are also often selected based on model fit statistics such as Akaike information criterion (AIC) and Bayesian information criterion (BIC) as in the case of forward selection. Randomized algorithms were proposed as an alternative approach that reduce the computational resource requirement and provide faster solutions than deterministic methods (Frieze et al., 2004; Sarlos, 2006; Achlioptas and Mcsherry, 2007; Liberty et al., 2007; Drineas et al., 2008; Ailon and Chazelle, 2009; Clarkson and Woodruff, 2017; Tropp et al., 2017). These methods provide approximations to the exact solutions in less time by trading accuracy for efficiency in solving high-dimensional problems (Musco, 2018). In randomized algorithms, features are selected or extracted randomly based on some probability distribution. Choosing a well-suited probability distribution ensures that the approximations are of high quality.

**FIGURE 1:**
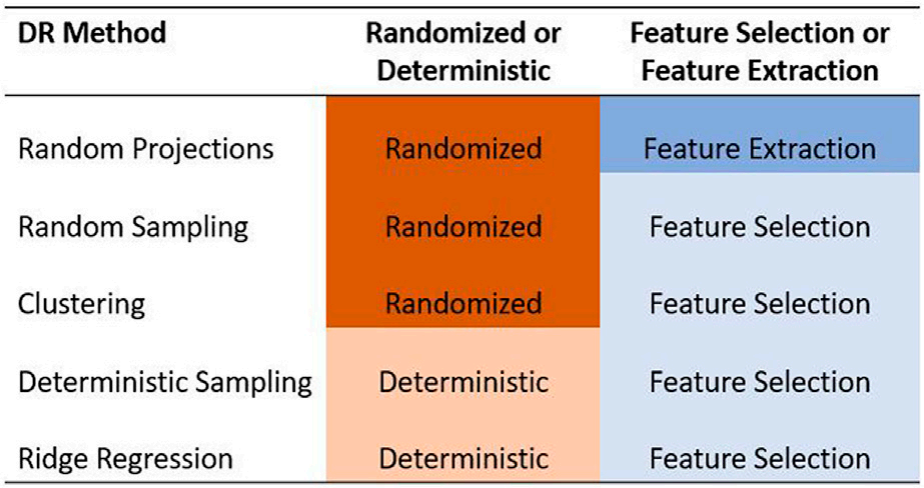
Summary of the five dimensionality reduction methods used in this paper. The methods are categorized as feature selection/feature extraction and as randomized/deterministic.

In this paper, we focused only on the feature selection and feature extraction approaches to DR. We examined the ability of five methods to reduce the dimensionality of the predictor data set in GS. Specifically, we compared the random projection algorithm proposed by Ailon and Chazelle (2009) to four feature selection algorithms based on random sampling (Boutsidis et al., 2009), deterministic sampling (Papailiopoulos et al., 2014), ridge regression (Hoerl and Kennard, 1970), and clustering (Sneath and Sokal, 1973). Random sampling and random projection methods are often referred to together as random sketching methods. Out of these five methods, we used a randomized approach to three of them - random projection, random sampling, and a clustering based feature selection algorithm. We compared these to two deterministic algorithms based on deterministic sampling and ridge regression. The five methods are summarized in Figure 1.

### 2.1 Random sketching

In order to understand the need for a random sketching algorithm, let us consider the simple example of linear regression. Suppose we have a **X** ∈ *R* ^*n*×*d*^ full rank matrix of predictor variables and a response vector **y** of length *n*. The least squares estimates can be computed as,

$$\hat{\beta }={\left(X^{\prime }X\right)}^{-1}X^{\prime }y.$$

Thus, we need the Gram matrix **X**′**X** and **X**′**y** to compute the solution. The computation of **X**′**X** requires *O* (*nd* ^2^) time and **X**′**y** requires *O* (*nd*) time. When *n* > *d*, this solution is easy to calculate and is very popular in practice. But, when *n*, *d* or both are large—as they tend to be in many modern data sets—this computational time can be practically prohibitive.

Random sketching is a popular method to reduce the computational complexity of this problem. Instead of using the full data set (**X**, **y**), we can use a carefully constructed sketch $\left(\tilde{X},\tilde{y}\right)$ to solve for the least squares coefficients. We define $\tilde{X}=SX$ and $\tilde{y}=Sy$ , where **S** is a randomly generated “sketching matrix” of size *r* × *n*, *r* ≪ *n*. The least squares solution will then be given by,

$$\hat{\beta_{s}}={\left({\tilde{X}}^{\prime }\tilde{X}\right)}^{-1}{\tilde{X}}^{\prime }\tilde{y},$$

where $\hat{\beta_{s}}$ refers to the sketched solution. The cost of computing this solution reduces to *O* (*rd* ^2^). Our two primary goals for a sketched solution are to ensure that the approximate solution is close to the original and that the computational time is significantly reduced. The careful construction of the sketching matrix **S** helps us achieve both these goals. The Johnson-Lindenstrauss (JL) Lemma (Johnson and Lindenstrauss, 1984) plays a crucial role in random sketching algorithms because it states that a set of points in a high-dimensional space can be embedded into a space of lower dimension where the distances between the points are nearly the same. The manner in which **S** is constructed leads to the two major classes of sketching algorithms - random projection and random sampling. We will describe these two algorithms in the next section.

### 2.2 Random projection

Random projection algorithms form one of the major classes of random sketching algorithms. Random projection algorithms “uniformize” the non-uniformity structure present by rotating the matrix to a basis where the uniform random sampling of features is nearly optimal. Random projection can be viewed as the process of dimensionality reduction to preserve the pairwise distances between observations.

**FIGURE 2:**
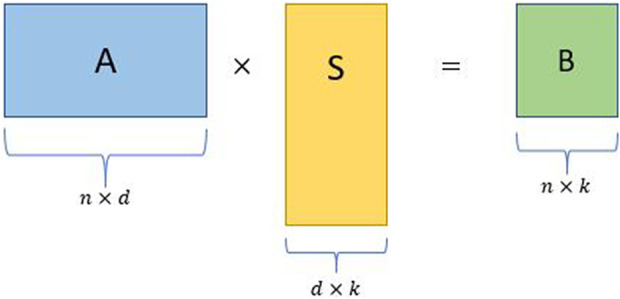
Schematic for a simple random projection where ***A*** is an input matrix, ***S*** is a random projection matrix, and ***B*** is the sketched matrix.

Random projection produces matrices formed by a small number of linear combinations of all of the original features. The linear combinations are formed by pre-multiplying the features with a randomly generated coefficients matrix, hence the name “random” projection. The resulting compressed matrix can be used as a substitute in computations, thereby reducing the computational complexity of the problem at hand. Given below is a simple random projection algorithm (Figure 2):

• Consider an input matrix **A** ∈ *R* ^*n*×*d*^ with *d* ≫ *n* without loss of generality.
• Construct a *d* × *k* random projection matrix **S**, where *k* ≪ *n*.
• Obtain the sketched matrix **B** = **AS**, where **B** is a *n* × *k* matrix.

Let us consider the example of performing the Singular Value Decomposition (SVD) of **A**. Using the original matrix **A**, the exact computation of SVD takes *O* (*nd* ^2^) time. Instead, if we settle for an approximate SVD of **A**, we can compute the SVD of **B** in place of **A**. The SVD computation on the smaller matrix **B** takes only *O* (*nk* ^2^) time even with the simple algorithm presented above. This example illustrates the motivation for using random projection to reduce the dimensionality of large matrices.

Two questions come to mind quite naturally when looking at the above mentioned approach. How do we choose a suitable **S** to obtain good approximations? For what values of *r* will this algorithm produce good results? These two questions have been extensively studied over the last couple of decades and have led to several candidates for the projection matrix **S** and corresponding *r* values. We summarize some popular sketching matrices proposed in previous works:

• **Gaussian Sketch:** One of the first sketches proposed for random projection by Sarlos (2006). The sketch **S** was formed by sampling each element from a Gaussian distribution **S**_*ij*_ ∼ *N* (0, 1/*r*), where **S**_*ij*_ refers to the element in the *i*th row and *j*th column of matrix **S**.
• **Rademacher Matrix:** Achlioptas and Mcsherry (2007) proposed a simpler sketching matrix where each element of the matrix **S** is a random variable taking { + 1, −1} with equal probability. Further, they also proved that a sparse matrix with 2/3 of the entries replaced with 0 satisfies the Johnson-Lindenstrauss property. This modification was an important development, as the random matrix **S** becomes a sparse matrix and leads to faster computations.
• **FJLT:** Ailon and Chazelle (2009) came up with the concept of fast Johnson-Lindenstrauss transforms (FJLT). The sketching matrix was generated as a product of three matrices **S** = *PHD*, where *P* is a *r* × *n* sub-sampling matrix, *H* is a *n* × *n* dimension Hadamard matrix, and *D* is a *n* × *n* random diagonal matrix with entries taking values { + 1, −1} with equal probability. A Hadamard matrix is a square matrix with elements either { + 1, −1} and all the rows are orthogonal.
• **CW Sketch:** The Clarkson and Woodruff (2017) sketch is also a sparse matrix formed as a product of two independent random matrices **S** = Γ*D*, where Γ is a *r* × *n* random matrix with only one element of each column set to +1 and *D* is a *n* × *n* random diagonal matrix with entries taking values { + 1, −1} with equal probability. Thus, **S** will be a sparse random matrix with one non-zero entry in each of its columns.

If computing the lower-dimension projection was so computationally expensive that not much performance improvement was gained on the whole, the whole exercise becomes futile. Thus, there has been significant research into building projection mappings that will efficiently implement the random projection. Sparsification was a popular tool to reduce the number of computations performed during projection, as seen with the Rademacher matrix and FJLT approaches. In this paper, we used the FJLT method, also known as the subsampled randomized Hadamard transform (SRHT), to implement the random projection method as it supports fast computation and requires a modest amount of storage compared to other methods. Further details about the FJLT method can be found in the Supplementary Materials S1.

Like any feature extraction based DR method, the random projection method is based on the linear combinations of all the original features. The newly created features may not be interpretable in the data scale. Even worse, they may have no practical meaning at all. For this reason, feature selection is an attractive alternative approach to feature extraction as a means of dimensionality reduction. A subset of the original features is picked using various strategies in feature selection. This allows for a straightforward interpretation of results. In the next sections, we explore four feature selection algorithms, starting with the random sampling algorithm, the second approach to randomized sketching algorithms.

### 2.3 Random sampling

Random sampling is another randomized approach to forming lower-dimension approximation matrices. While random projection addresses the non-uniformity by “uniformizing” the structure through rotations, random sampling algorithms build an importance probability to address the non-uniformity. The random sampling approach involves sampling a small number of features that represent the whole set of features. Leverage scores can be interpreted as a measure of influence the data points have on related computations and hence can be viewed as a metric to define the non-uniformity in the original matrix. Given a matrix **A** ∈ *R* ^*n*×*d*^, let *V*′ denote the matrix containing the top right singular vectors of **A**. Then, the statistical leverage score of the *i*th column of **A** is defined as $l_{i}={\left\|V_{\left(i\right)}^{\prime }\right\|}_{2}^{2}$ for *i* = 1, 2, *…* , *d*, where $V_{\left(i\right)}^{\prime }$ is the *i*th column of the matrix *V*′. Since, $\sum_{i=1}^{n}l_{i}=\|V^{\prime }{\|}^{2}=d$ , we can define a probability distribution over the columns of **A** given by *p*_*i*_ = *l*_*i*_/*d*. We will refer to this probability distribution as the importance probability distribution, which measures the relative importance of columns in the matrix. It provides a probability distribution based on which the random sampling can be carried out while accounting for the non-uniformity structure of the original matrix.

The computational bottleneck for using the importance probability distribution lies in its dependence on the computation of the orthogonal basis for the original input matrix. Drineas et al. (2012) provided an algorithm to compute the relative-error approximate leverage scores *l*_*i*_ instead of computing the exact statistical leverage scores. Their contribution was a key development in the area of random sampling algorithms. We used their algorithm as the basis for implementing the random sampling algorithm in the genomic prediction problem.

We now describe the random sampling algorithm presented in Drineas et al. (2012) along with drawing the attention of the reader to the salient properties and contributions. Consider a matrix **A** ∈ *R* ^*n*×*d*^ with *d* ≫ *n* and let *V*′ be the corresponding right singular matrix of **A**. Principally, we are interested in approximating the statistical leverage scores *l*_*i*_ of the columns of **A**, which are then used to construct the importance probability distribution.

$$l_{i}={\left\|V_{\left(i\right)}^{\prime }\right\|}_{2}^{2}={\left\|e_{i}^{\prime }\;V^{\prime }\right\|}_{2}^{2}$$ (1)

where *e*_*i*_ is the *i*th standard basis vector. The computation of the orthogonal matrix *V*′ takes *O* (*n* ^2^ *d*) time, which is the bottleneck. Since *V*′ can also be seen as any orthogonal basis for the column space of **A**, it follows that *V*′*V* = *AA* ^+^ where ^+^ is the Moore-Penrose inverse. From this, we can redefine the statistical leverage scores as,

$$l_{i}={\left\|e_{i}^{\prime }\;V^{\prime }\right\|}_{2}^{2}={\left\|e_{i}^{\prime }\;V^{\prime }V\right\|}_{2}^{2}={\left\|e_{i}^{\prime }\;AA^{+}\right\|}_{2}^{2}.$$ (2)

The computational complexity of calculating the leverage scores according to Eq. 2 involves computing the pseudo-inverse **A** ^+^ and performing the matrix multiplication of **A** and **A** ^+^. We apply random projection to overcome both these complexities by performing the computations and finally obtaining the approximate leverage scores ${\tilde{l}}_{i}$ .

Instead of computing **A** ^+^, we find a smaller matrix that approximates **A** and find the corresponding Moore-Pensore inverse of the smaller matrix. Subsampled Randomized Hadamard Transform (SRHT) is used to derive the smaller matrix as it preserves the structure **A** by rotating **A** to a random basis where all the rows have an equal influence and uniformly samples rows from that basis. If $\Pi_{1}\in R^{r_{1}\times n}$ is a *ϵ*-FJLT matrix for *V*′, then Π_1_ **A** is the approximation of **A**. Then Eq. 2 becomes,

$${\hat{l}}_{i}={\left\|e_{i}^{\prime }\;A{\left(\Pi_{1}A\right)}^{+}\right\|}_{2}^{2}.$$ (3)

While computing the product **AA** ^+^ takes *O* (*nd* ^2^), the computation of $A{\left(\Pi_{1}A\right)}^{+}$ takes *O* (*ndr*_1_) time. This is not efficient since *r*_1_ > *d*. Since only the Euclidean norms of the rows of $A{\left(\Pi_{1}A\right)}^{+}$ are required, the dimensionality of this matrix can be reduced by using a *ϵ*-JLT for the rows of $A{\left(\Pi_{1}A\right)}^{+}$ . Suppose $\Pi_{2}\in R^{r_{1}\times r_{2}}$ is an *ϵ*-JLT, then $A{\left(\Pi_{1}A\right)}^{+}\Pi_{2}$ is a randomized sketching of **AA** ^+^. Then we can compute the approximate statistical leverage scores as

$${\tilde{l}}_{i}={\left\|e_{i}^{\prime }\;A{\left(\Pi_{1}A\right)}^{+}\Pi_{2}\right\|}_{2}^{2}.$$ (4)

Drineas et al. (2012) showed that for any error parameter *ϵ* ∈ (0, 0.5] and any arbitrary matrix **A** of size *n* × *d* with *n* ≫ *d*, the expression

$$|l_{i}-{\tilde{l}}_{i}|\leq \epsilon l_{i}$$ (5)

holds for all *i* = 1, 2, *…* , *n*.

This result can be extended without loss of generality for *d* ≫ *n* case as well. Ma et al. (2015) investigated the approximation quality for several combinations of *r*_1_ and *r*_2_ through simulation studies. They found that *r*_1_ does not significantly impact the correlation between approximate and exact leverage scores but running time increases linearly with *r*_1_. On the other hand, the correlations between approximate and exact leverage scores increase rapidly with increasing *r*_2_, but *r*_2_ does not impact running time. Thus, they concluded that a combination of small *r*_1_ and large *r*_2_ would result in high-quality approximations with a short run time.

### 2.4 Deterministic sampling

Feature selection is also known as the column subset selection problem (CSSP) in matrix theory and linear algebra. Jolliffe (1972) proposed one of the first column subset selection algorithms. The algorithm involved deterministic sampling of the columns of the matrix based on ordered leverage scores. While the algorithm led to favorable dimensionality reduction in many practical applications, they did not provide theoretical guarantees on the quality of the approximation and hence it was not widely used for a long time. Drineas et al. (2008) developed the randomized counterpart to the deterministic sampling algorithm that employs a sampling probability distribution based on the leverage scores. They proved that their algorithm produces a matrix *C* that satisfies $\left\|A-CC^{+}A\right\|\leq \left(1+\epsilon \right)\left\|A-A_{k}\right\|$ with constant probability and hence guaranteed the approximation quality of their algorithm. Here, *A*_*k*_ is the best rank-k approximation obtained from SVD and *c* = *O* (*k* log  *k*/*ϵ* ^2^) is the number of columns in *C*.

The randomized algorithm gives a ‘near-optimal’ approximation of the matrix, but may not be computationally as efficient as the deterministic algorithm. Papailiopoulos et al. (2014) developed theoretical derivations for the approximation errors of the deterministic sampling algorithm provided by Jolliffe (1972). They proved that if the ordered leverage scores *l*_*i*_ follow a steep enough power-law decay, the deterministic algorithm performs equally or better than the randomized algorithm. Furthermore, suppose the leverage scores follow a steep power-law decay. In that case, the number of columns chosen by the deterministic algorithm is similar to or fewer than the randomized counterpart as proposed by Drineas et al. (2008). They showed the utility of the power-law decay assumption by providing several examples of real data sets where the leverage scores followed a power-law decay. Papailiopoulos et al. (2014) also emphasized that while their theoretical analysis was performed for the power-law decay model, other models for the leverage scores could be developed.

We now summarize the deterministic algorithm presented in Papailiopoulos et al. (2014). The deterministic algorithm can be described in three steps:

1. Compute the top-*k* right singular vectors **V**_*k*_ of **A** using SVD of **A**.
2. Calculate the leverage scores $l_{i}^{\left(k\right)}$ , where the superscript refers to the choice of *k* in the SVD. Reorder the leverage scores in a decreasing order.
3. Select *c* columns of **A** that correspond to the top *c* leverage scores such that their sum is greater than some stopping threshold *θ*, $\sum_{i=1}^{c}l_{i}^{\left(k\right)}>\theta$ . The choice of *θ* controls the quality of the approximation.

The deterministic sampling algorithm requires the implementation of SVD to compute the leverage scores. Hence, the time complexity of the algorithm is given by *O* (*nd* min (*n*, *d*)). The resulting matrix from the deterministic sampling algorithm guarantees a bound on the approximation error with regard to the CSSP. Implementing the deterministic algorithm for the genomic prediction can be seen as a pre-processing step. Given a large genomic information matrix, we use the deterministic algorithm to create a compressed matrix that represents the whole matrix well.

The deterministic sampling algorithm implementation mimics the random sampling implementation with an ordered sampling approach instead of a randomized sampling, based on the leverage scores. In this work, we also evaluated clustering and penalized regression approaches for DR. Since these methods are well established and widely popular, we do not present them in great detail.

### 2.5 Clustering

Clustering is the process of grouping a set of objects in such a way that objects in the same group are more similar to each other than to objects in different groups, called clusters. Grouping objects when the data are labeled is a trivial task and is often referred to as supervised classification (Jain et al., 1999). But, often we are presented with data with no labeling available. Clustering was developed as a tool to deal with problems where the objective was to group unlabeled objects into meaningful collections. Because of the absence of labels, clustering is also called as unsupervised classification.

The general scheme of clustering is to start with *n* objects and sort them into *K* groups based on some similarity measure such that the intra-group similarity is high and the inter-group similarity is low. Several categorizations of the clustering algorithms are available, such as partitional and hierarchical algorithms.

Partitional clustering divides the set into non-overlapping subsets (clusters) such that each object is present only in one cluster. Typically, partitioning clusters produce clusters by optimizing some criterion function to produce optimal solutions (Hartigan, 1975). K-means, the most popular partitional algorithm, is an algorithm where the objective is to minimize the sum of the squares of the distances from the objects to the centroid of the cluster. K-means algorithm ensures that there are always exactly *k* clusters at the end of the process, with each cluster containing at least one item. While the algorithm is efficient and easy to implement, it is prone to issues such as the need for globular clusters and the need for uniform cluster sizes. The K-means algorithm and its shortcomings are presented in greater detail in the Supplementary Materials S1. Some of these drawbacks can be overcome by using a hierarchical clustering approach instead.

#### 2.5.1 Hierarchical clustering algorithm

Hierarchical clustering is the process of creating a set of nested clusters arranged into a tree or dendrogram structure. Hierarchical clustering does not require a determination of the number of clusters *k* prior to the clustering process, as opposed to the k-means clustering. The nested structure provides flexibility of choosing the number of clusters based on the dendrogram as well as domain expertise (Jain et al., 1999). There are two possible directions of clustering under hierarchical clustering: agglomerative (bottom-up) and divisive (top-down). In this paper, we focus only on the agglomerative hierarchical clustering approach. The merging of clusters to form the hierarchy is determined by clustering metrics which define the similarity among the clusters. There are several clustering metrics available in the literature such as single-linkage, complete-linkage, average-linkage, and Ward’s method. We presented details about each of these clustering metrics in the Supplementary Materials S1.

Single-linkage is not the preferred metric due to its susceptibility to produce elongated clusters. Complete-linkage is avoided because of its inability to retain large clusters. Between average-linkage and Ward’s minimum variance method, there is no real distinguishing factor. Since Ward’s method can be compared easily to the objective function in k-means, we picked the Ward’s method as our metric of choice for the hierarchical clustering approach.

Hierarchical clustering creates a nested clustering structure, often represented by a dendrogram, which allows the user to create any number of clusters by choosing the appropriate height to cut the dendrogram. One of our objectives was to study the trend in prediction accuracy as a function of the input data sizes. Thus, we are interested in creating reduced data sets of different sizes. To obtain *t* data sets of different sizes, the K-means algorithm needs to be run *t* times. On the other hand, hierarchical clustering needs to be performed only once to determine the hierarchy. The *t* different sized marker data sets can then be created by cutting the dendrogram *t* times at appropriate heights.

### 2.6 Shrinkage methods

Variable selection is the process of choosing a subset of the explanatory variables to explain a response variable. Variable selection helps in making models easier to interpret, reducing noise introduced by redundant variables, and reducing the size of the data set for faster computations. When the number of variables is very large, traditional subset selection methods such as “best” subset selection are computationally infeasible. Step-wise selection methods were proposed as an alternative to reduce the computational load. A major drawback of the traditional and step-wise subset selection methods is the discrete nature of the variable selection, i.e., the variables are either retained or discarded. This leads to unstable variable selection, where a small change in data can lead to large change in the subset selected (Breiman, 1996).

Shrinkage methods were developed to address the shortcomings of the subset selection methods. These methods are also known as regularization or penalized methods. They work on the principle of imposing a constraint term that penalizes for model complexity. Shrinkage methods help in variable selection as well as improving the model’s prediction performance through the bias-variance trade-off. In other words, shrinkage methods may provide solutions that have lower variance and higher bias, but ultimately leading to better prediction accuracy according to the mean squared error (MSE).

In this paper, we investigate the shrinkage methods as a tool for variable selection. We use the coefficients of the predictors, obtained from the shrinkage methods, as a form of ranking of the variables for selection purposes.

Ridge regression uses all the *d* predictors in the final model. The algorithm shrinks all coefficients towards 0 through the *L*_2_ penalty, but does not set any of them exactly equal to zero. Hence, none of the predictors are removed from the final model. On the other hand, LASSO is a shrinkage method that applies an *L*_1_ penalty on the regression coefficients. Due to the nature of the *L*_1_ penalty, LASSO performs both shrinkage and automatic variable selection (Tibshirani, 1996). In other words, the penalty term not only shrinks the coefficients towards 0, it sets some of the coefficients to 0. When *d* > *n*, LASSO selects at most *n* variables (Zou and Hastie, 2005). Further, LASSO selects only one variable at random from a group of high correlated variables, often the case with genomic data. This can be a significant drawback in situations where selecting one of the variables from the group implies that all other variables are important as well because LASSO selects only one and discards the rest of the variables in the group.

Zou and Hastie (2005) proposed a new shrinkage method called the elastic net to overcome the problems presented by LASSO while retaining the advantages of LASSO. Elastic net can be viewed as a combination method involving both ridge regression and LASSO (Zou and Hastie, 2005). Elastic net allows for variable selection and also allows for group selection of variables, acting as an ideal combination of ridge regression and LASSO. It is appropriate for scenarios where *d* > *n*. We describe these three shrinkage methods - LASSO, ridge regression, and elastic net - along with their respective advantages and drawbacks in the supplementary section. Further details about the shrinkage methods can also be found in James et al. (2013).

The disadvantages presented by the LASSO algorithm, especially in context of genomic data makes it unsuitable for this study. Elastic net would be the ideal shrinkage algorithm for dimensionality reduction in practice. Unfortunately, it does not provide control on the number of variables selected in the final model. To answer all the objectives of the study, we needed fine control on the number of variables selected by each reduction method to help us compare the DR approaches to one another. Ridge regression performs shrinkage on the coefficients associated with the variables, but does not perform variable selection of any kind. Thus, we picked ridge regression as the shrinkage method of choice for this study.

In ridge regression, the penalty parameter has to be estimated separately. There are several methods for estimating the most appropriate penalty parameter *λ*. The most popular and reliable method is cross-validation. We can choose a range of *λ* values, compute the cross-validated error for each value of *λ* and pick the *λ* corresponding to the smallest cross-validation error (James et al., 2013).

## 3 Implementation of methods

In this paper, we used five different DR methods as pre-processing step to genomic prediction models. We implemented the methods to reduce the dimensionality of the genomic data to help reduce the computational resource requirements such as memory, storage, and time. In this section, we present details about the implementation of each of the five DR methods on the genomic data to create data sets of differing sizes to evaluate the trends in prediction as the function of the dimensionality.

### 3.1 Implementation of the random projection algorithm

**FIGURE 3:**
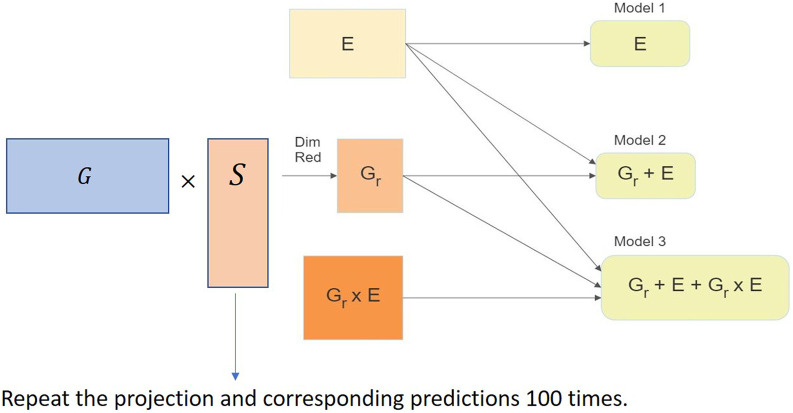
Implementation of the random projection algorithm for dimensionality reduction of the genomic data set in the genomic prediction problem.

We used the *RaProR* package (Geppert et al., 2019) in **R** (R Core Team, 2020) to compute the random projection. The package was built based on theorems and results in Geppert et al. (2017), which provides details about the algorithms implemented to compute the projection. We used the SRHT projection for our random projection implementation in this study. The implementation of the random projection algorithm for dimensionality reduction of the genomic marker data set can be visualized in Figure 3 and can be summarized as follows:

1. Compute projection matrices with a predefined number of columns *k*.
2. Multiply the projection matrix with original marker data to obtain the reduced matrix *X* of size *n* × *k*.
3. Use the reduced matrix to compute the genetic relationship matrix as *G* = *XX*′/*d*, where *G* denotes the genetic relationship matrix, as the input for genomic prediction models and obtain predictions in different cross-validation schemes (described in Sections 4.1 and 4.2). The genetic relationship matrix describes the relationship between individuals, and captures the population structure.
4. Repeat steps 2–4 100 times to remove bias in the prediction results.

### 3.2 Implementation of the random sampling algorithm

We used the statistical leverage scores to calculate the importance probability distribution required for the random sampling algorithm. We followed the two-stage algorithm presented by Boutsidis et al. (2009) to implement the random sampling algorithm. The implementation of the random sampling algorithm for dimensionality reduction of the marker data set for genomic prediction is as follows:

1. Compute the approximate leverage scores as defined in Drineas et al. (2012).
2. Use the approximate leverage scores to define the importance sampling distribution for the columns of the input marker matrix *X*.
3. Randomly sample a predefined number of columns *k* according to the importance sampling distribution to form reduced matrices of different sizes.
4. Use the reduced matrix *X* to compute the genetic relationship matrix *G* = *XX*′/*d* as the input for the prediction models and obtain predictions in different cross-validation schemes (described in Sections 4.1 and 4.2).
5. Repeat steps 3–4 100 times to remove sampling bias from the prediction accuracy results.

The advantage of this random sampling algorithm is the computation of the approximate leverage scores instead of the exact scores, effectively reducing computation time.

### 3.3 Implementation of clustering for dimensionality reduction

This section describes our approach to applying dimensionality reduction to genomic data sets using clustering. The R package “*fastcluster*” (Müllner, 2013) was used for fast implementation of the hierarchical clustering algorithm. Once the dendrogram was created, the cuts were made to form the *k* clusters. The cut height is determined by the R package to ensure that the user-defined *k* number of clusters are created. The implementation of the clustering algorithm for dimensionality reduction of the genomic data set can be summarized as follows:

1. Perform agglomerative hierarchical clustering using “*fastcluster*” to determine the hierarchy.
2. Form *k* clusters from the hierarchy by cutting appropriately.
3. Sample one feature randomly from each of the *k* clusters as the representative of that cluster.
4. The sampled features form the reduced data set of size *k* and will be used for the genomic prediction models.
5. Repeat the sampling from the clusters and the following model implementation 100 times to remove sampling bias in the prediction accuracy results.

### 3.4 Implementation of the deterministic sampling algorithm

The deterministic sampling algorithm implementation mimics the random sampling implementation without the randomized sampling based on the leverage scores. The deterministic sampling algorithm can be summarized as follows:

1. Compute the approximate leverage scores as defined in Drineas et al. (2012).
2. Arrange the leverage scores in decreasing order.
3. Pick the top *k* leverage scored columns to form the reduced matrices.
4. Use these reduced matrices as the input for the genomic prediction models.

### 3.5 Implementation of ridge regression for dimensionality reduction

We used the “*glmnet*” (Simon et al., 2011) package in **R** to implement ridge regression. Given below is the implementation of the ridge regression algorithm for dimensionality reduction of the genomic data set for genomic prediction:

1. Implement a ridge regression model with the standardized features corresponding to the marker information.
2. Use the coefficients estimated from the ridge regression as a measure of importance of the features.
3. Order the features by their respective regression coefficients.
4. Pick the top *k* features to form the reduced data set for the genomic prediction models.

## 4 Data and genomic prediction models

All the methods were applied to a chickpea data set collected by the International Chickpea Screening Nursery (ICSN) of ICRISAT (Roorkiwal et al., 2016). The lines were phenotyped for three seasons (2012–13, 2013–14, and 2014–15) at two locations (ICRISAT, Patancheru and IARI, New Delhi) under different water regimes (normal-rainfed, irrigated, or late-sown), which resulted in nine environments (unique season-location-water combinations). Phenotypic data on eight traits were collected: 100 Seed Weight (100-SDW measured in grams), Biomass (BM measured in grams), Days to 50% Flowering (DF measured number of days), Days to Maturity (DM measured in number of days), Harvest Index (HI measured in %), Plant Height (PH measured in centimeters), Number of Plant Stand (PS measured in number of plants) and Seed Yield (SY measured in grams). Since one of the most important trait for plant breeders is yield, in this paper, we evaluated the DR methods with the seed yield (SY) as the phenotype of interest. The calculations can be performed the same way for the other traits.

The original data set contained 315 lines phenotyped in nine environments, giving a total of 2,835 phenotypic yield observations. All of the 315 lines had corresponding genomic data with 26,817 markers each. After cleaning the data as described in the Supplementary Materials S1, the genomic data had 306 observations and 14,928 features, which could be viewed as a matrix of size 306 × 14,928. In the following section, we describe the models used for genomic prediction as well as the techniques used to evaluate the accuracy of the models.

### 4.1 Prediction models

In this work, we used the models proposed by Jarquín et al. (2014) to evaluate the predictive ability of reduced datasets. Specifically, we considered three models based on the input information in each model: either environmental and line information (E + L) only, or genomic information along with environment information (G + E), or genomic information with environmental information as well as their interactions (G + E + G×E) as the predictors.

Let the phenotypic trait be represented by *y*_*ijk*_ for the *k*th replicate for the *j*th line in the *i*th environment. Let the environmental effect be represented by *E*_*i*_ (*i* = 1, 2, … , *I*), the line effect be defined by *L*_*j*_ (*j* = 1, 2, …, *J*), the genetic effect be denoted by *g*_*j*_ (*j* = 1, 2, …, *J*), the interaction be denoted by *gE*_*ij*_ and the error term be represented as *ϵ*_*ijk*_ (*k* = 1, 2, …, *r*_*ij*_). The three models corresponding to the three scenarios mentioned above are given by:

$$y_{\mathrm{ijk}}=\mu +E_{i}+L_{j}+\epsilon_{\mathrm{ijk}},$$ (6)

$$y_{\mathrm{ijk}}=\mu +E_{i}+g_{j}+\epsilon_{\mathrm{ijk}},$$ (7)

$$y_{\mathrm{ijk}}=\mu +E_{i}+g_{j}+gE_{ij}+\epsilon_{\mathrm{ijk}},$$ (8)

where *μ* is the overall mean, $E_{i}∼N\left(0,\sigma_{E}^{2}\right)$ , $L_{j}∼N\left(0,\sigma_{L}^{2}\right)$ , $g∼N\left(0,G\sigma_{g}^{2}\right)$ , $gE∼N\left(0,\left[Z_{g}GZ_{g}^{\prime }\right]◦\left[Z_{e}Z_{e}^{\prime }\right]\sigma_{gE}^{2}\right)$ and $\epsilon_{\mathrm{ijk}}∼N\left(0,\sigma_{\epsilon }^{2}\right)$ ; $\sigma_{E}^{2},\sigma_{g}^{2}$ , and $\sigma_{\epsilon }^{2}$ are environment, genetic and residual variances, respectively. The variance component of the **gE** interaction is represented by $\sigma_{gE}^{2}$ . The incidence matrices for the effect of the genomic values and environment are **Z**_*g*_ and **Z**_*e*_, respectively. The genetic relationship matrix is **G**, computed as **G** = *XX*′/*d* where *X* is the centered and scaled molecular markers matrix and *d* is the number of SNPs. Finally, ◦ denotes the Schur product (element by element product) between two matrices.

Using the dimensionality reduction methods, we reduce the size of the marker matrix (X) and thus the dimensionality reduction methods affect only the G + E (Eq. 7) and G + E + G × E (Eq. 8) models but not the baseline E + L model (Eq. 6).

### 4.2 Model assessment using cross-validation schemes

Three different cross-validation schemes were implemented to assess the predictive ability of the models in different scenarios. These are scenarios that breeders might be interested in since these mimic the situations they face in their breeding programs. The performance of the prediction models was assessed by measuring the Pearson correlations (Waldmann, 2019) between the observed phenotypic values and the predicted genomic estimated breeding values within environments. The three different cross-validations can be summarized as the prediction of lines in a new unobserved environment (**CV0**), the prediction of new untested lines in environments (**CV1**), and the prediction of lines that were observed in some environments but not observed in other environments (**CV2**).

**CV0** refers to the cross-validation that evaluates the ability of the models to predict the performance of lines in a new unobserved environment. In effect, we performed a *k*-fold cross-validation in which we left out the observations from the observed target environment in each fold and used the other observations from the other (*k* − 1) environments as the training set. We computed the correlations between the observed and predicted values within each environment. This correlation quantified a model’s ability to predict the performance of all the lines in a new environment.

**CV1** refers to the cross-validation that evaluates the ability of the models to predict the performance of untested lines in all environments. For CV1 a five-fold cross-validation scheme was used where we randomly selected 20% of the lines as the testing group and left out all the observations corresponding to these lines from all environments. We used the observations from the other 80% of the lines as the training set to build the prediction models. Then, we predicted the trait values for the lines left out across all environments. This process of creating folds randomly and performing predictions was repeated 20 times. Finally, we computed the correlations between the observed and predicted values within each environment and averaged them across the 20 runs to obtain the average correlations.

**CV2** refers to the cross-validation that evaluates the ability of the models to predict the performance of lines that were tested in some environments but not tested in other environments. For CV2, the phenotypic observations were randomly partitioned into five subsets without regard for the lines or environment. Four subsets were combined and used for training the models, and the remaining subset was used as a test set. The process was repeated 20 times, just as described in CV1, to obtain average correlations.

## 5 Dimensionality reduction in genomic prediction

We investigated five DR methods in this paper. We performed feature selection or feature extraction for each method to create reduced dimensional marker data sets of 26 different sizes based on the number of markers present. The number of markers ranged from 200 to 14,928 (the full marker data set). We set the number of markers as fixed across all methods to compare the dimensionality reduction methods and their prediction ability at each size. For the three randomized dimensionality reduction methods, 100 data sets were generated at each size, and prediction results from the 100 data sets were averaged and set as the prediction accuracy of that size. This was done to overcome any bias that the random selection process may have introduced. For each reduced data set, we implemented the three predictions models (Eqs 6–8) and evaluated each model using the three cross-validation schemes described in the previous section. CV1 and CV2 cross-validation schemes were run 20 times on each set due to the randomization introduced during the fold creation. Taking into consideration all the different methods, sizes, randomization, prediction models, and cross-validation schemes, we ran over 965,000 combinations in this study.

## 6 Results

This study focused on two objectives. The primary objective was to evaluate the merit of using dimensionality reduction methods as a pre-processing step in genomic prediction. We used five dimensionality reduction methods to present dimensionality reduction as an effective pre-processing step in genomic prediction. We compared their reduction capabilities to find which methods work better for genomic prediction across different prediction models and cross-validation schemes. Second, we studied the trends in prediction accuracy of reduced data sets as a function of their size.

**FIGURE 4:**
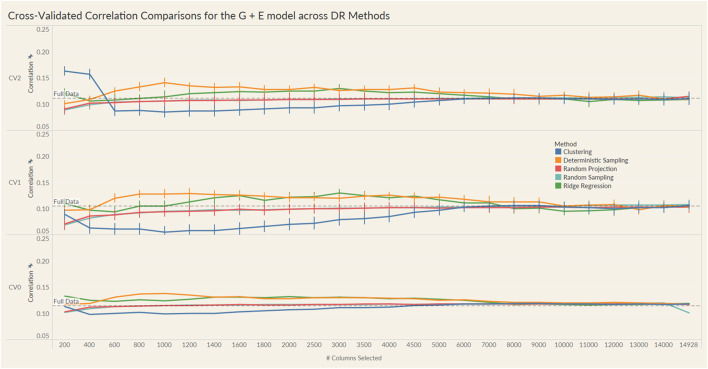
Prediction accuracy for the seed yield trait of a chickpea population consisting of 306 genotypes tested in nine environments for the G + E model under the three cross-validation schemes (CV0, CV1, CV2) across 26 different genomic information sizes. Standard errors are depicted at each size.

**FIGURE 5:**
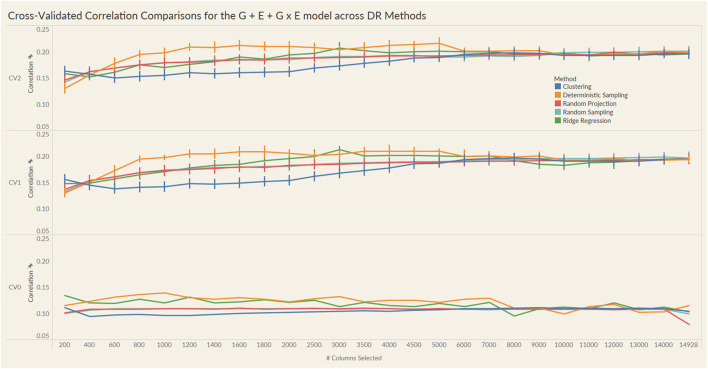
Prediction accuracy for the seed yield trait of a chickpea population consisting of 306 genotypes tested in nine environments for the G + E + GxE model under the three cross-validation schemes (CV0, CV1, CV2) across 26 different genomic information sizes. Standard errors are depicted at each size.

The results from these models are summarized in the plots in Figures 4, 5. For each model and cross-validation combination, the correlation results for the full unreduced SNP data are referenced using a grey horizontal line.

Irrespective of the model and CV scheme, all dimensionality reduction methods required only a fraction of the total input markers to obtain maximum correlation. In addition, we observed a plateauing of correlation values as the number of markers selected increased for all methods. Thus, the number of markers required to achieve maximum correlation may be an inappropriate measure to evaluate the reduction capability of the method. Instead, we considered a 95% of the maximum correlation as our metric to evaluate the reduction methods. For instance, for the CV1 scheme in the G × E model, the random projection algorithm achieved the maximum correlation of 0.195 with all 14,928 input markers. However, the method achieved a 95% maximum correlation of 0.185 with just 3,000 input markers. This significantly reduces the number of input markers for similar correlation values. In fact, for all the dimensionality reduction methods, fewer than 40% of the input markers were required to achieve a 95% max correlation value. These results are summarized in Table 1. Furthermore, for the deterministic sampling and ridge regression based reduction, the highest correlation was achieved by a reduced data set rather than using the whole data set in all three models, indicating the presence of noise in the data.

**TABLE 1:** Number of markers selected by each dimensionality reduction (DR) method to obtain 95% of the highest correlation for the three prediction models (G + E + GxE, G + E, E + L) under the three cross-validation schemes (CV0, CV1, CV2).

Pred. Model	CV	DR Method	# Cols	Correlation
G + E + GxE	CV0	Clustering	200	0.105
		DetSampling	800	0.132
		RanProj	400	0.104
		RanSampling	400	0.104
		Ridge	200	0.127
	CV1	Clustering	4,500	0.185
		DetSampling	1,200	0.199
		RanProj	3,000	0.185
		RanSampling	4,000	0.189
		Ridge	3,000	0.202
	CV2	Clustering	4,500	0.188
		DetSampling	1,200	0.205
		RanProj	3,000	0.189
		RanSampling	3,000	0.191
		Ridge	2,500	0.197
G + E	CV0	Clustering	200	0.113
		DetSampling	600	0.131
		RanProj	400	0.112
		RanSampling	800	0.114
		Ridge	200	0.126
	CV1	Clustering	6,000	0.101
		DetSampling	800	0.123
		RanProj	1,600	0.098
		RanSampling	5,000	0.103
		Ridge	1,600	0.124
	CV2	Clustering	200	0.155
		DetSampling	1,000	0.134
		RanProj	2000	0.109
		RanSampling	1800	0.108
		Ridge	1,600	0.124
E + L	CV0		14,928	0.089
	CV1		14,928	-0.098
	CV2		14,928	0.045

Second, no one reduction method had the best reduction capability across all prediction models and CV combinations. For instance, for the CV2 scheme of the G × E model, deterministic sampling required only 1,200 markers in the input data to achieve 95% of the maximum correlation compared to the 4,500 required by clustering. On the other hand, for the CV2 scheme of the G + E model, clustering required only 200 input markers to attain 95% of the maximum correlation compared to the 1,000 required by deterministic sampling. Random projection and random sampling methods were very similar in terms of prediction accuracy values across all matrix sizes by model by cross-validation combinations. All the reduction methods had similar prediction accuracies within the model and CV combination, which reiterates the utility of dimensionality reduction regardless of the DR method used.

## 7 Discussion

Modern plant breeding programs combine genomic information with phenotypic performance data to select favorable lines. Early genomic selection models included the line, environment, phenotypic and genomic information to predict the performance of lines. Genetic information, environmental factors, and their interactions affect complex traits such as yield. Hence, the development of models that allowed for this genotype by environment interactions improved the genomic prediction accuracy. The improvements in genotyping technology combined with the reducing cost have led to the generation of genomic data of enormous sizes that are often high-dimensional. While current genomic selection models can handle these high-dimensional data, there are questions about their efficiency. Further, including information on hundreds of thousands of potentially unrelated markers in the genomic prediction models could negatively impact the prediction accuracy of the trait of interest. Lastly, there is a computational resource cost that must be taken into account. Prediction models with larger input sizes require much greater computational resources to run, both in terms of hardware and time. We proposed dimensionality reduction as a mechanism to address all of these concerns.

In this study, we used a chickpea data set. Chickpea is the second largest produced food legume crop in the world (Roorkiwal et al., 2016). Its high protein content makes it a valuable source of protein in several cultures worldwide, especially in vegetarian diets. Implementation of GS methods helps breeding programs reduce breeding cycle time and improve the rate of genetic gains (Roorkiwal et al., 2018) by allowing breeders to select lines using genomic marker data before performing field trials. With the recent improvements in the high-throughput genotyping technologies, millions of markers are available for several hundred chickpea lines. GS has been adept at accessing these large data sets to predict the performance of lines. However, further advances in this area will yield larger marker data sets, which could overwhelm the current GS methods and the available computational resources. In addition, other high-dimensional data such as high-dimensional weather covariates and high-throughput phenotyping information are increasingly more available in breeding programs. While we did not consider such data in this study, the inclusion of such data could create computational bottlenecks. These reasons present a need to develop methods that effectively handle large data sets and use the additional data available to improve prediction accuracy.

The key contribution of this work was to propose using dimensionality reduction in genomic prediction analyses and show its utility using a hand-picked subset of all available methods. For example, we explored the possibility of using randomized algorithms for dimensionality reduction with the help of primitive implementations. Several more sophisticated randomized algorithms could improve dimensionality reduction, which can be explored in future works. Our results act as a proof of concept that future researchers can use to explore various dimensionality reduction methods and identify the best method for their breeding data. Our results clearly indicate the need to integrate dimensionality reduction methods into genomic selection to reduce computational resource requirements, improve the prediction process, and select the best performing lines in any breeding program.

## Data Availability

The original contributions presented in the study are included in the article/Supplementary Material, further inquiries can be directed to the corresponding author.
