# Supplementary material for: Evaluating dimensionality reduction for genomic prediction
Source: Front Genet. 2022 Oct 14;13:958780. doi: 10.3389/fgene.2022.958780 (PMC9614092; doi:10.3389/fgene.2022.958780)
Supplement: Supplementary file 1 [file DataSheet1.PDF]

Supplementary Material

This supplementary provides additional detail about each of the dimensionality reduction (DR) methods. First, we review some key definitions and notations that will be used in the rest of the supplementary section. Following that, we present details about DR method. Next, we elaborate on the data cleaning procedure as well as the cross-validations schemes used for model evaluation.

Before we present the relevant literature for each of the DR methods, we review some key linear algebra definitions and notations. We use e_j to denote the j-th standard basis vector in R^n, i.e., e_j is a vector with its j-th entry equal to 1 and all other entries equal to 0. Throughout this paper, $||\cdot||_2$ is used to denote the spectral norm. We also use $||\cdot||_F$ to denote the Frobenius norm. The spectral and Frobenius norms of a matrix $\mathbf{A} \in R^{m\times n}$ are defined as $||\mathbf{A}||_2 = \sup_{x\in R^n, x\neq 0} \frac{|Ax|}{|x|}$ and $||\mathbf{A}||_F = \sqrt{\sum_{i=1}^{m}\sum_{j=1}^{n} a_{ij}^2}$, respectively. Here a_{ij} are elements in matrix $\mathbf{A}$ and $|\cdot|$ is the Euclidean vector norm defined as $|x| = \sqrt{\sum_{i=1}^{n} x_i^2}$.

Statistical leverage scores have been an integral part of data analysis for many decades. They have been used for outlier detection in regression analysis (Hoaglin and Welsch, 1978; Chatterjee, 1988) and as a measure of leverage or influence a data point has (Velleman and Welsch, 1981), and hence have found application in randomized matrix algorithms (Drineas et al., 2012, 2008; Mahoney and Drineas, 2009; Drineas et al., 2011). Given a matrix $\mathbf{A} \in R^{n\times d}$, let V' denote the matrix containing the top right singular vectors of $\mathbf{A}$. Then, the statistical leverage score of the i-th column of $\mathbf{A}$ is defined as $l_i = \left\|V'_{(i)}\right\|_2^2$ for $i = 1, 2, \ldots, d$, where $V'_{(i)}$ is the i-th column of the matrix V'.

1 RANDOM SKETCHING

1.1 Johnson-Lindenstrauss Lemma and its extensions

Dimensionality reduction involves some form of mapping data from a high-dimensional space to a lower dimensional space in such a manner that the information from the original data is retained. The existence of a mapping that can approximately preserve pairwise distances while embedding data from a high-dimensional space into a lower-dimensional space was guaranteed by a lemma by Johnson and Lindenstrauss (1984) and is commonly known as the Johnson Lindenstrauss Lemma (JL Lemma). JL Lemma is a fundamental component of all random projection algorithms and hence we present a brief summary of the lemma and its extensions before discussing random projection.

Johnson and Lindenstrauss (1984) showed that n points in an Euclidean space R^d can be projected onto a $r = O(\log n/\epsilon^2)$ dimensional space without distorting any pairwise distances of the n points by more than a factor of $(1 \pm \epsilon)$ for any $\epsilon \in (0, 1/2]$. Johnson and Lindenstrauss provided this lemma as a tool to prove extensions of Lipschitz mapping into a Hilbert space. But due to its distance preserving nature, it has become a popular tool in dimensionality reduction.

JL Lemma: For any $\epsilon \in (0, 1/2]$ and any set of n points $x_1, x_2, \ldots, x_n$ in R^d, there exists a projection map $f : R^d \to R^r$, where $r = O(\log n/\epsilon^2)$. Then, for all $i,\ j \in \{1, 2, \ldots, n\}$,

$$(1-\epsilon)\|x_i - x_j\|_2^2 \leq \|f(x_i) - f(x_j)\|_2^2 \leq (1+\epsilon)\|x_i - x_j\|_2^2. \tag{S1}$$

Johnson and Lindenstrauss provided a lengthy technical proof using geometric approximation. Their main idea was summarized succinctly by Fedoruk et al. (2018):

- Project a set of points $x_1, x_2, \ldots, x_n$ in R^d onto a random r- dimensional space.
- The expected length of each r-dimensional vector is $\sqrt{r/d}$ times the length of the original vectors.
- Multiplying each projection by scaling factor of $\sqrt{d/r}$ yields r-dimensional vectors which are similar in length to the original d-dimensional vectors.
- If we choose a tolerance limit ϵ, then with non-zero probability each length is preserved within the chosen tolerance limit.

The JL Lemma has had numerous improvements and extensions over time. The improvements were two-pronged: improvements in the bounds for r and improvements to the efficiency of $f(\cdot)$. The original proof by Johnson and Lindenstrauss suggested the lower bound for r as $O(\log n/\epsilon^2)$. Frankl and Maehara (1988) improved the lower bound to $r = O(9 \log n/(\epsilon^2 - 2\epsilon^3/3))$. Further, they also provided a method to find a suitable mapping. JL Lemma only proves the existence of a mapping function $f(\cdot)$, but does not provide a way of finding one. Dasgupta and Gupta (2003) relied on probabilistic techniques to improve the lower bound results to $r = O(4 \log n/(\epsilon^2/2 - 2\epsilon^3/3))$. Their proof is also considered significantly simpler than the original proof by Johnson and Lindenstrauss (1984). Both these papers retained the definition of the random projection from the original paper, summarized below (Ailon and Chazelle, 2009) :

- Spherical symmetry: For any orthogonal matrix A, A and $f(A)$ have the same distribution.
- Orthogonality: The rows of $f(\cdot)$ are orthogonal to each other.
- Normality: The rows of $f(\cdot)$ are unit-length vectors.

Improvements in the efficiency of $f(\cdot)$ were made through relaxing the assumptions listed above. Har-Peled et al. (2012) showed that the JL Lemma could be satisfied even without the orthogonality and normality conditions being met. They proposed a projection matrix R where each element of the matrix was sampled from the $N(0, 1/d)$ distribution.

1.2 Random Projections

We summarize the thought process followed in Ailon and Chazelle (2009). Suppose we have an arbitrary vector $\mathbf{x}$. If $\mathbf{x}$ was uniformly distributed, uniform random sampling and re-scaling can lead to a good estimate of the l_2-norm of $\mathbf{x}$. But, often $\mathbf{x}$ may not be uniformly distributed and thus uniform random sampling will lead to poor estimates of the l_2-norm. Uncertainty principle states that the more dense a function $f(\cdot)$, the more spread out its Fourier transformation and vice-versa. As a consequence of this, if $\mathbf{x}$ is sparse then its Fourier transform $F\mathbf{x}$ cannot be too sparse where F is a Fourier transformation. By definition, a Hadamard transform is a multi-dimensional Fourier Transform. Hence, $H\mathbf{x}$ also cannot be too sparse, where H is a Hadamard transform. Despite this, $H\mathbf{x}$ could still be sparse and so we re-randomize $H\mathbf{x}$ with a cost-efficient rotation such as a diagonal matrix D with its elements taking the values $\{+1, -1\}$ with equal probability. Finally, we use a sub-sampling matrix P of size $r \times n$ which helps us sample from $HD\mathbf{x}$. Thus, we have the final form of the FJLT given by $PHD\mathbf{x}$. Due to the presence of the Hadamard matrix, this projection is also known as the Subsampled Randomized Hadamard Transform (SRHT).

Some of the key points to note from SRHT are:

- Since D is diagonal, $D\mathbf{x}$ can be computed in $O(n)$ time.
- H is applied to the n-dimensional vector $D\mathbf{x}$ in $O(n \log n)$ time.
- P is applied to an n-dimensional vector in $O(r)$ time.

- Finally, the SRHT of a $n \times d$ matrix can be computed in $O(nd \log r)$ time.

The Hadamard-based sketching scheme, aka the FJLT, was particularly important for fast implementations of the random projection algorithms. FJLT was first proposed by Ailon and Chazelle (2009) and was later applied to randomized algorithms in the form of SRHT (Sarlos, 2006; Drineas et al., 2011). The SRHT sketch was analyzed in detail by Tropp (2011). Tropp also presented a simpler proof that the SRHT satisfies the JL property. Boutsidis and Gittens (2013) improved upon this work and provided bounds for r which have low dependence on n. They also extended the results by applying SRHT for the approximation of matrix multiplication.

From (Woodruff, 2014; Geppert et al., 2017; Ahfock et al., 2019), we can summarize a comparison of the sketches in terms of the sketching time and the corresponding value for r. The results are summarized in Table S1.

2 CLUSTERING

Clustering is the process of grouping a set of objects in such a way that objects in the same group are more similar to each other than to objects in different groups, called clusters. Grouping objects when the data are labeled is a trivial task and is often referred to as supervised classification (Jain et al., 1999). But, often we are presented with data with no labeling available. Clustering was developed as a tool to deal with problems where the objective was to group unlabeled objects into meaningful collections. Because of the absence of labels, clustering is also called as unsupervised classification.

Clustering has seen immense interest in recent decades through its various applications in pattern and object recognition, recommender systems, and machine learning. It was first introduced in anthropology by Driver and Kroeber (1932) where they grouped cultures from different tribes of Polynesia and the Americas based on the presence or absence of traits such as matrilineage, sinew-backed bow, twined weaving, ridged houses, etc. Over the years clustering has been applied as a classification tool in many fields such as social science, psychology, biology, marketing, medicine, etc (Hartigan, 1975; Punj and Stewart, 1983; Jiang et al., 2004; Clatworthy et al., 2005; Sutherland et al., 2012). Clustering is also used for detecting anomalies in the data, for identifying degree of similarity of objects, and for organizing and summarizing data.

2.1 Partitional Clustering Algorithm

Partitional clustering divides the set into non-overlapping subsets (clusters) such that each object is present only in one cluster. Typically, partitioning clusters produce clusters by optimizing some criterion function to produce optimal solutions (Hartigan, 1975). K-means, the most popular partitional algorithm, is an algorithm where the objective is to minimize the sum of the squares of the distances from the objects to the centroid of the cluster. K-means algorithm ensures that there are always exactly k clusters at the end of the process, with each cluster containing at least one item.

The general form of the k-means algorithm is as follows:

1. Choose the number of cluster, k.
2. Randomly assign k out of n items as cluster centroids.
3. Assign all the remaining $(n - k)$ items in the collection to their nearest cluster based on distance to the centroid.
4. Recompute the cluster centroids based on the current cluster assignment.
5. Reassign items to their nearest cluster based on distance to centroids.
6. Repeat steps 4-5 until no items change cluster assignments, or an iteration threshold is met.

K-means clustering is a very efficient and easy algorithm to apply. However, it may not be a suitable algorithm in certain cases:

- **Non-globular cluster:** The solution to the k-means clustering is obtained by minimizing the within-cluster sum of squares. This is minimized when the clusters are globular and when the clusters are well separated from each other. Thus, applying k-means to non-globular shapes leads to results that are not optimal. Non-globular clusters can be visualized as in Figure 1a.
- **Non-uniform cluster sizes:** The cluster sizes are expected to be more or less similar to each other. K-means algorithm is not suitable when there is large variation in the cluster sizes from one cluster to the next. This issue is depicted in Figure 1b.
- **Presence of outliers:** K-means algorithm is sensitive to outliers. The algorithm updates the centroids of the clusters by taking the average of all the points in the cluster. The presence of outliers will pull the centroid towards the outliers and lead to misclassification of data points into the wrong clusters. There are several solutions in the literature to perform k-means in the presence of outliers (Dave and Krishnapuram, 1997; Gan and Ng, 2017; Jiang et al., 2016; Hautamäki et al., 2005).

Some of the drawbacks such as the need for globular clusters and the need for cluster sizes to be similar can be overcome by using a hierarchical clustering approach rather than a partitional clustering algorithm. We expand on the hierarchical clustering method in the next section.

2.2 Hierarchical Clustering

Hierarchical clustering is the process of creating a set of nested clusters arranged into a tree or dendrogram structure. Hierarchical clustering does not require a determination of the number of clusters k prior to the clustering process, as opposed to the k-means clustering. The nested structure provides flexibility of choosing the number of clusters based on the dendrogram as well as domain expertise (Jain et al., 1999). There are two possible directions of clustering under hierarchical clustering: agglomerative (bottom-up) and divisive (top-down). In agglomerative clustering, each object is a cluster by itself initially and the most similar clusters are paired together successively to form a hierarchy. In divisive clustering, we start with the entire set of objects as one cluster and recursively divide each cluster into sub-clusters based on dissimilarity. Both these approaches lead to a hierarchy among objects which can be represented by a dendrogram. A typical agglomerative hierarchical clustering scheme is given below:

1. Start with each item in its own cluster.
2. Compute all similarity between all pairs of clusters.
3. Merge the two clusters that are most similar to each other based on the clustering metric.
4. Repeat the process until only one cluster is remaining.

2.3 Clustering Metrics

There are several metrics that can be used to compute the similarity or dissimilarity between clusters. In this paper, we describe four of the most popular metrics: single-linkage (Sneath and Sokal, 1973), complete-linkage (King, 1967), average-linkage (Hastie et al., 2001) and Ward's method (Murtagh, 1983; Ward, 1963).

Single-linkage, also known as minimum linkage, method computes all pairwise distance between the elements within two clusters, and considers the smallest of these distances as a linkage criterion. The single-linkage algorithm is versatile in applications, but often tends to produce elongated clusters. To merge two clusters using single-linkage, only one object of the cluster needs to be close to the other cluster. Thus,

this could lead to chaining and elongated clusters. Single-linkage also does not perform well if there is noise between clusters. In other words, single-linkage is not suitable if the clusters are not well separated.

Complete-linkage method considers the largest value (i.e., maximum value) of these distances as the distance between the two clusters. Complete-linkage method tends to produce more compact clusters and is also less susceptible to outliers and noise. It produces well-bounded compact clusters compared to single-linkage method, but tends to break large clusters into smaller ones. Jain and Dubes (1988) observed that complete-link is often the sensible option in most applications.

Average-linkage method computes all pairwise distances between elements in two clusters and takes the average of all distances as the distance between the two clusters. Average-linkage algorithm also performs well in the presence of noise between clusters. But like the complete-linkage algorithm, it tends to produce globular clusters.

Ward's method, or Ward's minimum variance method, computes the distance between two clusters as the increase in the total within-clusters sums of squares when two clusters are merged. The two clusters whose union leads to the smallest increase in the sum of the squares are merged together. Ward's method has the same disadvantages as complete-linkage, whereby it favors globular clusters.

3 SHRINKAGE METHODS

Variable selection is the process of choosing a subset of the explanatory variables to explain a response variable. Variable selection helps in making models easier to interpret, reducing noise introduced by redundant variables, and reducing the size of the data set for faster computations. For these reasons, variable selection proves to be an important step in the analysis of high-dimensional data where the implementation and interpretation of models are made difficult due to the large number of variables present.

When the number of variables is very large, traditional subset selection methods have significant drawbacks. The best subset selection method involves fitting separate models for each possible combination of the p predictors. For a problem with p predictors, there are 2^p possible subset selections. The task of finding the "best" subset is practically unfeasible as p increases. Stepwise selection methods are an alternative to the best-subset-selection method. They fit only a restricted set of models as opposed to the best-subset-selection method. Forward and backward stepwise selection are the two popular stepwise selection algorithms. Both evaluate only $1 + p(p + 1)/2$ models compared to the 2^p models that are evaluated by best-subset-selection. Even though both these algorithms do not guarantee finding the "best" model containing a subset of the p predictors, they are known to perform well in practice (James et al., 2013). In high dimensional problems where $p > n$, backward selected cannot be used because of the initialization of the algorithm by fitting the full model. Forward selection can be used even when $p > n$ as its initialization depends on fitting the null model containing no predictors.

Another drawback of the traditional subset selection methods is the discrete nature of the variable selection, i.e., the variables are either retained or discarded. This leads to unstable variable selection, where a small change in data can lead to large change in the subset selected (Breiman, 1996). Shrinkage methods were developed to address the shortcomings of the subset selection methods. These methods are also known as regularization or penalized methods. They work on the principle of imposing a constraint term that penalizes for model complexity. Shrinkage methods help in variable selection as well as improving the model's prediction performance through the bias-variance trade-off. In other words, shrinkage methods may provide solutions that have lower variance and higher bias, but ultimately leading to better prediction accuracy according to the mean squared error (MSE).

3.1 Ridge Regression

Consider a standard linear regression model,

$$y_i = \beta_0 + \sum_{j=1}^{p} \beta_j X_{ij} + \epsilon$$

where y_i is the response value for the i-th observation, X_{ij} represents the j-th predictor for the i-th observation, and β_j is the coefficient of the j-th predictor. The coefficients β_j can be estimated as the values that minimize the residual sum of squares (RSS) function,

$$RSS = \sum_{i=1}^{n} \left(y_i - \beta_0 - \sum_{j=1}^{p} \beta_j x_{ij} \right)^2 . \tag{S2}$$

The estimates obtained by minimizing the RSS are known as the ordinary least squares (OLS) estimates. The OLS estimates are reliable only if the predictors are orthogonal. Further, if we have a high-dimensional problem with $p > n$, then OLS does not have a solution. Ridge regression was proposed by Hoerl and Kennard in 1970 as a solution to problems where OLS estimates are unreliable (Hoerl and Kennard, 1970). While OLS estimates are unbiased, ridge regression estimates are biased. But, the increase in bias is compensated by a decrease in variance and results in estimates with smaller MSE.

Ridge regression is similar to least squares regression, but the estimates are obtained by minimizing a different objective function. In OLS, the coefficients are estimated by minimizing the RSS function. In ridge regression, the coefficients β_j are estimated by minimizing a penalized residual sum of squares,

$$\sum_{i=1}^{n} \left(y_i - \beta_0 - \sum_{j=1}^{p} \beta_j x_{ij} \right)^2 + \lambda \sum_{j=1}^{p} \beta_j^2 = RSS + \lambda \sum_{j=1}^{p} \beta_j^2, \tag{S3}$$

where $\lambda \geq 0$ is a penalty parameter. $\lambda \sum_{j=1}^{p} \beta_j^2$ is called the penalty term and we note that it takes an L_2 penalty form. λ controls the amount of shrinkage of the parameters β_j. The larger the penalty parameter, the greater the amount of shrinkage and the greater the coefficients are shrunk towards 0. When $\lambda = 0$, the ridge regression estimates are equal to the OLS estimates. At $\lambda = 0$, the variance is high and the bias is low. As λ increases, the variance reduces substantially but the bias increases only marginally. Thus, ridge regression provides equally or more accurate predictions compared to OLS regression. Further, if $p > n$, OLS does not provide unique solution whereas ridge regression can perform well by exploiting the bias-variance trade-off.

In ridge regression, the penalty parameter has to be estimated separately. There are several methods for estimating the most appropriate penalty parameter λ. The most popular and reliable method is cross-validation. We can choose a range of λ values, compute the cross-validated error for each value of λ and pick the λ corresponding to the smallest cross-validation error (James et al., 2013). Another method is to pick λ by an automated procedure as proposed by Hoerl et al. (1975). They proposed selecting the value of the penalty parameter as $\lambda = \frac{rs^2}{\sum_{j=1}^{p} \hat{\beta}_j^2}$, where r is the number of parameters in the model, s^2 is the RSS from the least squares estimation, and $\hat{\beta}_j$ are the least square estimates of the regression coefficients.

Ridge regression uses all the p predictors in the final model. The term $\lambda \sum_{j=1}^{p} \beta_j^2$ shrinks all coefficients towards 0, but does not set any of them exactly equal to zero. Hence, none of the predictors are removed from the final model. This can be perceived as a disadvantage in the context of the variable selection problem. In this paper, our focus is on reducing the dimensionality of the data using variable selection. Both subset-selection and dimension reduction methods lead to reducing the number of predictor variables used in the final model. Thus, another shrinkage method called Least Absolute Shrinkage and Selection Operator (LASSO) was proposed by Tibshirani (1996) that overcomes this disadvantage and allows the shrinkage to be 0.

3.2 LASSO

LASSO is a shrinkage method that applies an L_1 penalty on the regression coefficients. In ridge regression, an L_2 penalty was applied. Due to the nature of the L_1 penalty, LASSO performs both shrinkage and automatic variable selection (Tibshirani, 1996). In other words, the penalty term not only shrinks the coefficients towards 0, it sets some of the coefficients to 0. Thus, LASSO inherently 'eliminates' the predictors from the final model that have coefficients 0.

In ridge regression, the coefficients are estimated by minimizing a penalized residual sum of squares with a L_2 penalty. With LASSO, the coefficients β_j are estimated by minimizing a penalized residual sum of squares with an L_1 penalty:

$$\sum_{i=1}^{n} \left(y_i - \beta_0 - \sum_{j=1}^{p} \beta_j x_{ij} \right)^2 + \lambda \sum_{j=1}^{p} |\beta_j| = RSS + \lambda \sum_{j=1}^{p} |\beta_j|, \tag{S4}$$

where $\lambda \geq 0$. When the penalty parameter λ is sufficiently large, some of the coefficient estimates are set to be exactly equal to 0. A detailed explanation of why LASSO sets some coefficients to 0 while ridge regression does not, can be found in Tibshirani (1996) and James et al. (2013). Thus, LASSO performs variable selection. As with the ridge regression, when $\lambda = 0$ the LASSO estimates are equivalent to the OLS estimates. The penalty parameter λ has to be estimated separately, similar to ridge regression. The cross-validation method of searching for optimal value of λ works with LASSO as well and is the most popular one.

LASSO has its own set of disadvantages. When $p > n$, LASSO selects at most n variables (Zou and Hastie, 2005). Further, LASSO selects only one variable at random from a group of high correlated variables. This can be a significant drawback in situations where selecting one of the variables from the group implies that all other variables are important as well because LASSO selects only one and discards the rest of the variables in the group. Zou and Hastie (2005) proposed a new shrinkage method called the elastic net to overcome the problems presented by LASSO while retaining the advantages of LASSO.

3.3 Elastic Net

Elastic net can be viewed as a combination method involving both ridge regression and LASSO (Zou and Hastie, 2005). In elastic net, the coefficients β_j are estimated by minimizing a penalized residual sum of squares with an elastic net penalty term:

$$\sum_{i=1}^{n}\left(y_i - \beta_0 - \sum_{j=1}^{p}\beta_j x_{ij}\right)^2 + \lambda\sum_{j=1}^{p}\left((1-\alpha)|\beta_j| + \alpha\beta_j^2\right)$$
$$= RSS + \lambda\sum_{j=1}^{p}\left((1-\alpha)|\beta_j| + \alpha\beta_j^2\right), \quad \text{(S5)}$$

where $\alpha = \frac{\lambda_2}{\lambda_1+\lambda_2}$. Here, $\lambda_1 \geq 0$ and $\lambda_2 \geq 0$ are penalty parameters. $\left((1-\alpha)|\beta_j| + \alpha\beta_j^2\right)$ is called the elastic net penalty term. When $\alpha = 0$, elastic net is equivalent to the ridge regression and when $\alpha = 1$, elastic net is equivalent to LASSO.

Elastic net allows for variable selection and also allows for group selection of variables, acting as an ideal combination of ridge regression and LASSO. It is appropriate for scenarios where $p > n$. Similar to ridge regression and LASSO, appropriate values for the penalty parameters have to be estimated separately. The cross-validation method performs well for elastic net as well. It should be noted that instead of searching among a range of possible values for the penalty parameter, elastic net requires searching among a grid of values corresponding to the two penalty parameters present.

4 DATA CLEANING PROCEDURE

The original data set contained 315 lines phenotyped in nine environments, giving a total of 2835 phenotypic yield observations. All of the 315 lines had corresponding genomic data with 26,817 markers each. The following steps were taken in the data-cleaning process:

1. Removed all lines (rows in the data set) that had more than 90% missing information in their genotypic data. This led to the deletion of 9 lines, leaving 306 lines in the data set.
2. Removed the corresponding lines in the phenotypic data across all environments.
3. Removed all markers (features) with the Minor Allele Frequency (MAF) less than 0.05. There were no features removed in this step.
4. Removed all markers with more than 50% missing values, leaving 14928 markers in the genotypic data set.
5. Imputed the remaining missing values in the marker data by taking the average of the rest of the entries in their respective columns.
6. Centered and scaled the genotypic data matrix.

5 SUPPLEMENTARY FIGURES AND TABLES

Sketch	Running Time	Sketch size (r)
Gaussian	$O(ndr)$	$O(\{d + \log(1/\delta)\}\epsilon^{-2})$
Rademacher	$O(ndr)$	$O(\{d + \log(1/\delta)\}\epsilon^{-2})$
SRHT	$O(nd\log r)$	$O(\{d\log(d/\delta)\}\epsilon^{-2})$
CW	$O(nd)$	$O(d^2/\epsilon^2\delta)$

Table S1. Sketching time and necessary sketching size r for different sketching schemes. The necessary sketch size r refers to the minimum size such that the resulting random projection matrix satisfies the JL property with a probability of at least $(1-\delta)$.

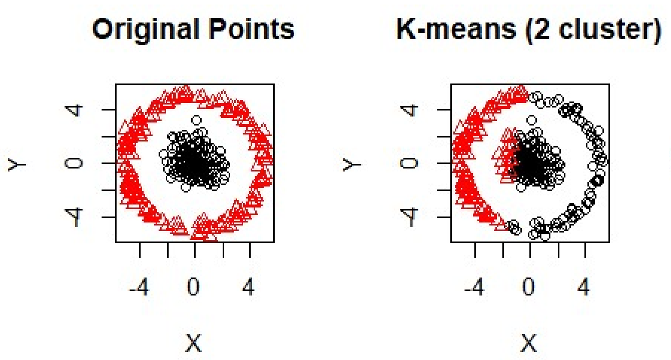

(1a) Non-globular clusters

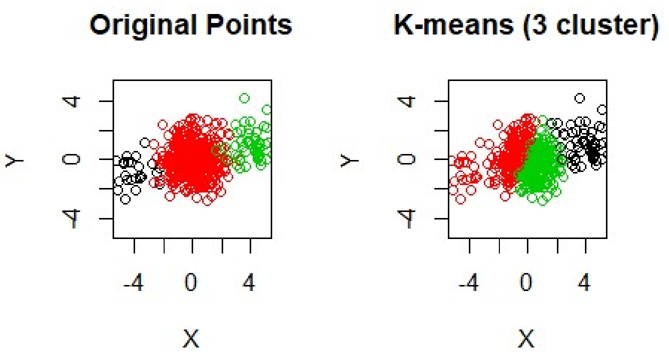

(1b) Non-uniform cluster sizes

Figure S1: Limitations of K-means algorithm

REFERENCES

Ahfock, D., Astle, W. J., and Richardson, S. (2019). Statistical properties of sketching algorithms. *arXiv:1706.03665 [stat]* ArXiv: 1706.03665

Ailon, N. and Chazelle, B. (2009). enThe Fast Johnson–Lindenstrauss Transform and Approximate Nearest Neighbors. *SIAM Journal on Computing* 39, 302–322. doi:10.1137/060673096

Boutsidis, C. and Gittens, A. (2013). enImproved matrix algorithms via the Subsampled Randomized Hadamard Transform. *arXiv:1204.0062 [cs, math]* ArXiv: 1204.0062

Breiman, L. (1996). enHeuristics of instability and stabilization in model selection. *Annals of Statistics* 24, 2350–2383. doi:10.1214/aos/1032181158. Publisher: Institute of Mathematical Statistics

Chatterjee, S. (1988). *Sensitivity Analysis in Linear Regression* (USA: John Wiley & Sons, Inc.)

Clatworthy, J., Buick, D., Hankins, M., Weinman, J., and Horne, R. (2005). The use and reporting of cluster analysis in health psychology: A review. *British Journal of Health Psychology* 10, 329–358. doi:https://doi.org/10.1348/135910705X25697

Dasgupta, S. and Gupta, A. (2003). enAn elementary proof of a theorem of Johnson and Lindenstrauss. *Random Structures & Algorithms* 22, 60–65. doi:https://doi.org/10.1002/rsa.10073. _eprint: https://onlinelibrary.wiley.com/doi/pdf/10.1002/rsa.10073

Dave, R. N. and Krishnapuram, R. (1997). Robust clustering methods: a unified view. *IEEE Transactions on Fuzzy Systems* 5, 270–293. doi:10.1109/91.580801. Conference Name: IEEE Transactions on Fuzzy Systems

Drineas, P., Magdon-Ismail, M., Mahoney, M. W., and Woodruff, D. P. (2012). Fast approximation of matrix coherence and statistical leverage. *The Journal of Machine Learning Research* 13, 3475–3506

Drineas, P., Mahoney, M. W., and Muthukrishnan, S. (2008). Relative-Error CUR Matrix Decompositions. *SIAM Journal on Matrix Analysis and Applications* 30, 844–881. doi:10.1137/07070471X

Drineas, P., Mahoney, M. W., Muthukrishnan, S., and Sarlós, T. (2011). enFaster least squares approximation. *Numerische Mathematik* 117, 219–249. doi:10.1007/s00211-010-0331-6

Fedoruk, J., Schmuland, B., Johnson, J., and Heo, G. (2018). enDimensionality reduction via the Johnson–Lindenstrauss Lemma: theoretical and empirical bounds on embedding dimension. *The Journal of Supercomputing* 74, 3933–3949. doi:10.1007/s11227-018-2401-y

Frankl, P. and Maehara, H. (1988). The johnson-lindenstrauss lemma and the sphericity of some graphs. *Journal of Combinatorial Theory, Series B* 44, 355–362. doi:https://doi.org/10.1016/0095-8956(88)

90043-3

Gan, G. and Ng, M. K.-P. (2017). enk -means clustering with outlier removal. *Pattern Recognition Letters* 90, 8–14. doi:10.1016/j.patrec.2017.03.008

Geppert, L. N., Ickstadt, K., Munteanu, A., Quedenfeld, J., and Sohler, C. (2017). enRandom projections for Bayesian regression. *Statistics and Computing* 27, 79–101. doi:10.1007/s11222-015-9608-z

Har-Peled, S., Indyk, P., and Motwani, R. (2012). ENApproximate Nearest Neighbor: Towards Removing the Curse of Dimensionality. *Theory of Computing* 8, 321–350. doi:10.4086/toc.2012.v008a014. Publisher: Theory of Computing Exchange

Hartigan, J. A. (1975). *Clustering Algorithms* (USA: John Wiley & Sons, Inc.), 99th edn.

Hastie, T., Tibshirani, R., and Friedman, J. (2001). *The Elements of Statistical Learning*. Springer Series in Statistics (New York, NY, USA: Springer New York Inc.)

Hautamäki, V., Drapkina, S., Kärkkäinen, I., and Kinnunen, T. (2005). Improving K-Means by Outlier Removal. vol. 3540, 978–987. doi:10.1007/11499145_99

Hoaglin, D. C. and Welsch, R. E. (1978). The Hat Matrix in Regression and ANOVA. *The American Statistician* 32, 17–22. doi:10.1080/00031305.1978.10479237. Publisher: Taylor & Francis _eprint: https://www.tandfonline.com/doi/pdf/10.1080/00031305.1978.10479237

Hoerl, A. E., Kannard, R. W., and Baldwin, K. F. (1975). Ridge regression:some simulations. *Communications in Statistics* 4, 105–123. doi:10.1080/03610927508827232. Publisher: Taylor & Francis _eprint: https://doi.org/10.1080/03610927508827232

Hoerl, A. E. and Kennard, R. W. (1970). enRidge Regression: Biased Estimation for Nonorthogonal Problems. *Technometrics* 12, 55–67. doi:10.1080/00401706.1970.10488634

Jain, A. K. and Dubes, R. C. (1988). *Algorithms for Clustering Data* (USA: Prentice-Hall, Inc.)

Jain, A. K., Murty, M. N., and Flynn, P. J. (1999). enData clustering: a review. *ACM Computing Surveys* 31, 264–323. doi:10.1145/331499.331504

James, G., Witten, D., Hastie, T., and Tibshirani, R. (2013). en*An Introduction to Statistical Learning*, vol. 103 of *Springer Texts in Statistics* (New York, NY: Springer New York). doi:10.1007/978-1-4614-7138-7

Jiang, D., Tang, C., and Zhang, A. (2004). Cluster analysis for gene expression data: a survey. *IEEE Transactions on Knowledge and Data Engineering* 16, 1370–1386. doi:10.1109/TKDE.2004.68

Jiang, L., Chen, H., Pinello, L., and Yuan, G.-C. (2016). GiniClust: detecting rare cell types from single-cell gene expression data with Gini index. *Genome Biology* 17, 144. doi:10.1186/s13059-016-1010-4

Johnson, W. and Lindenstrauss, J. (1984). Extensions of lipschitz maps into a hilbert space. *Contemporary Mathematics* 26, 189–206. doi:10.1090/conm/026/737400

King, B. (1967). Step-Wise Clustering Procedures. *Journal of the American Statistical Association* 62, 86–101. doi:10.2307/2282912. Publisher: [American Statistical Association, Taylor & Francis, Ltd.]

Mahoney, M. W. and Drineas, P. (2009). enCUR matrix decompositions for improved data analysis. *Proceedings of the National Academy of Sciences* 106, 697–702. doi:10.1073/pnas.0803205106

Murtagh, F. (1983). enA Survey of Recent Advances in Hierarchical Clustering Algorithms. *The Computer Journal* 26, 354–359. doi:10.1093/comjnl/26.4.354

Punj, G. and Stewart, D. W. (1983). Cluster analysis in marketing research: Review and suggestions for application. *Journal of Marketing Research* 20, 134–148. doi:10.1177/002224378302000204

Sarlos, T. (2006). enImproved Approximation Algorithms for Large Matrices via Random Projections. In *2006 47th Annual IEEE Symposium on Foundations of Computer Science (FOCS'06)* (Berkeley, CA: IEEE), 143–152. doi:10.1109/FOCS.2006.37

Sneath, P. H. A. and Sokal, R. R. (1973). EnglishNumerical taxonomy. The principles and practice of numerical classification. *Numerical taxonomy. The principles and practice of numerical classification.*

Sutherland, E. R., Goleva, E., King, T. S., Lehman, E., Stevens, A. D., Jackson, L. P., et al. (2012). Cluster analysis of obesity and asthma phenotypes. *PLOS ONE* 7, 1–7. doi:10.1371/journal.pone.0036631

Tibshirani, R. (1996). Regression shrinkage and selection via the lasso. *Journal of the Royal Statistical Society. Series B (Methodological)* 58, 267–288

Tropp, J. A. (2011). Improved analysis of the subsampled randomized Hadamard transform. *arXiv:1011.1595 [cs, math]* ArXiv: 1011.1595

Velleman, P. F. and Welsch, R. E. (1981). Efficient Computing of Regression Diagnostics. *The American Statistician* 35, 234–242. doi:10.1080/00031305.1981.10479362. Publisher: Taylor & Francis _eprint: https://www.tandfonline.com/doi/pdf/10.1080/00031305.1981.10479362

Ward, J. H. (1963). Hierarchical Grouping to Optimize an Objective Function. *Journal of the American Statistical Association* 58, 236–244. doi:10.2307/2282967. Publisher: [American Statistical Association, Taylor & Francis, Ltd.]

Woodruff, D. P. (2014). enSketching as a Tool for Numerical Linear Algebra. *Foundations and Trends® in Theoretical Computer Science* 10, 1–157. doi:10.1561/0400000060. ArXiv: 1411.4357

Zou, H. and Hastie, T. (2005). enRegularization and variable selection via the elastic net. *Journal of the Royal Statistical Society: Series B (Statistical Methodology)* 67, 301–320. doi:10.1111/j.1467-9868.2005.00503.x
